# Association Between a History of Contact Sport Participation and Higher Lifetime Mild Traumatic Brain Injury Burden Among Military Servicemembers and Veterans: A Long-term Impact of Military-relevant Brain Injury Consortium–Chronic Effects of Neurotrauma Consortium Prospective Longitudinal Study (LIMBIC-PLS)

**DOI:** 10.1177/23259671251407330

**Published:** 2026-06-01

**Authors:** Kelly C. Cheever, Rocio N. Norman, Samuel R. Walton, Clara E. Dismuke-Greer, William C. Walker, Monica Lawson, Alicia A. Swan, Guo Wei, Angela Presson, Huong Nguyen, Emre Umucu, Mary Jo Pugh

**Affiliations:** † Kinesiology Department, University of Texas at San Antonio, San Antonio, Texas, USA; ‡ Human Performance Research Interest Group, University of Texas at San Antonio, San Antonio, Texas, USA; § Department of Communication Sciences and Disorders, University of Texas Health Science Center, San Antonio, Texas, USA; ‖ Department of Physical Medicine and Rehabilitation, Virginia Commonwealth University School of Medicine, Richmond, Virginia, USA; ¶ Richmond Veterans Affairs (VA) Medical Center, Central Virginia VA Health Care System, Richmond, Virginia, USA; # Institute of Women's Health, Virginia Commonwealth University, Richmond, Virginia, USA; ** Health Economics Resource Center, VA Palo Alto Health Care System, Palo Alto, California, USA; †† Psychology Department, University of Texas at San Antonio, San Antonio, Texas, USA; ‡‡ Polytrauma System of Care, South Texas Veterans Health Care System, Department of Veterans Affairs, San Antonio, Texas, USA; §§ Department of Epidemiology, University of Utah, Salt Lake City, Utah, USA; ‖‖ Department of Public Health Services, University of Texas at El Paso, El Paso, Texas, USA; ¶¶ Research, Education, Academic, Center on Health Disparities, University of Texas at El Paso, El Paso, Texas, USA; ## Department of Medicine, Division of Epidemiology, Spencer Fox Eccles School of Medicine, University of Utah, Salt Lake City, Utah, USA; Investigation performed at University of Texas at San Antonio, San Antonio, Texas, USA

**Keywords:** mTBI, contact sport, military, long-term health

## Abstract

**Background:**

Both years of contact sport participation and years of military service are positively related to risk of sustaining multiple mild traumatic brain injuries (mTBIs). Little is known about the additive risk of mTBI exposure by combining premilitary contact sport participation with subsequent military service.

**Purpose:**

To assess the effect of premilitary contact sport participation on the odds of current and former military personnel sustaining mTBI(s) during their lifetime.

**Study Design:**

Cross-sectional study; Level of evidence, 3.

**Methods:**

This study analyzed data from 2651 individuals from the LIMBIC-PLS. The association between a history of contact sport participation and sustaining an mTBI pre–military service, during military service while not on deployment, or during deployment was estimated using odds ratios. The association between the total number of pre–military service, military service while not on deployment, and deployment-related mTBI events with history of contact sport participation (yes vs no) was estimated using incidence rate ratios (IRRs) and 95% confidence intervals. The authors adjusted those models for demographic factors and then repeated those analyses using years of contact sport exposure, categorized as 0 (referent), >0 to 5, >5 to 10, and >10 years.

**Results:**

In this cohort, 62% had a history of contact sport participation and 82% had a history mTBI. Individuals with a history of contact sport participation were more likely to have experienced an mTBI in their lifetime (OR, 1.26; 95% CI, 1.03-1.54; *P* = .023). This manifested only outside of military combat deployments. Specifically, a history of contact sport exposure was associated with a higher odds of an mTBI before military service (OR, 1.63; 95% CI, 1.39-1.91; *P* < .001) or a nondeployment mTBI (OR, 1.62; 95% CI, 1.37-1.91; *P* < .001) Moreover, a higher incidence rate of lifetime mTBI was observed as number of years of contact sport exposure increased.

**Conclusion:**

Similar to findings in civilians, these findings suggest that contact sport participation increases the likelihood of experiencing a single or multiple mTBI events among military personnel. These findings suggest that it would be appropriate to screen individuals’ mTBI history before joining the military to identify individual potential risks for concerns associated with multiple lifetime mTBI events.

Mild traumatic brain injury (mTBI) and its potential long-term neurological consequences pose a serious public health concern with impacts on tens of millions of people's social, economic, and academic well-being across the world each year. Two populations particularly vulnerable to these injuries are contact sport athletes and military personnel.^12,21^ While an estimated 1 in 10 Americans report a history of mTBI, this number is thought to be as high as 1 in 4 among athletes and 1 in 2 among military personnel.^5,7,21,25^ While a single mTBI can cause long-term alterations to an individual's health, evidence is mounting for deleterious cumulative effects and long-term consequences of sustaining multiple injuries across the lifespan.^5,15^ Even when termed “mild” on the spectrum of traumatic brain injury (TBI) severities, the compounding risk of potential alterations in the parenchyma and axonal network communication of the brain is proposed to be associated with long-term neurological consequences. These include issues such as depression,^10^ cognitive impairment,^9,18^ neurodegenerative disease,^8^ posttraumatic stress disorder,^21,22^ cognitive impairment,^4^ and chronic traumatic encephalopathy.^18,23^ While the risk of sustaining multiple mTBIs in a lifetime is relatively low among the general population, contact sport athletes and military personnel face an increased likelihood of experiencing recurrent mTBI events.^20,24^ Furthermore, the risk of experiencing multiple mTBI events has been demonstrated to rise independently with increased years of sport participation^2^ and military service, specifically combat deployment time.^17^

While both years of contact sport participation and years of military service are positively associated with the risk of sustaining multiple mTBI events,^2^ little is known about the cumulative risk for persons participating in contact sports throughout adolescence and later entering military service. The purpose of the present report was to determine the relative odds of having experienced a pre–military service mTBI between military personnel with and without a history of premilitary contact sport participation. Second, we sought to determine the relative incidence of mTBI events by accounting for years of contact sport exposure on the number of lifetime mTBI events between the 2 groups. We hypothesized that veterans and military servicemembers with a history of contact sport participation would have higher odds of having experienced at least 1 mTBI before military service compared to those without a history of contact sport participation. We further hypothesized that those with a history of contact sport participation would have a higher relative incidence of mTBI across their lifespan.

## Methods

### Design

The present study included a cross-sectional analysis using data captured during the initial (baseline) visit of the longitudinal, multicenter prospective longitudinal study (PLS) conducted by the Long-term Impact of Military-relevant Brain Injury Consortium–Chronic Effects of Neurotrauma Consortium (LIMBIC-CENC).^26,27^ The LIMBIC-CENC PLS was developed to address the long-term effects of neurotrauma in US military servicemembers and veterans. During the baseline visit, participants completed a comprehensive, multimodal assessment including structured interviews, questionnaires, comprehensive neurological and neuropsychological testing, and biometric measurements that have been previously described.^19^

### Participants

The PLS was conducted across 10 recruitment sites and had enrolled approximately 2680 participants who completed baseline assessments at the time of data extraction. The cohort is composed of active-duty military servicemembers, reservists, national guard members, and veterans. To be eligible, participants must have been previously deployed to at least 1 combat zone, have been at least 18 years of age at the time of the baseline visit, and have had no history of moderate or severe TBI, major neurological disorder, or psychiatric illnesses resulting in a significant long-term decrease in functional status (eg, schizophrenia or spinal cord injury).^26^ The study obtained approval from the institutional review boards of the participating facilities, and written informed consent was obtained from all participants before any procedures were conducted. For the present secondary data analysis study, 29 participants were excluded from the sample for the following reasons: 21 individuals had no stop date for contact sport, 5 had no military start date, and 3 were missing other pertinent data.

### Primary Outcome: mTBI Events

The history of mTBI events was obtained through a rigorous, standardized structured interview and record review process. Each participant's potential concussive events (PCEs) were cataloged using a modified version of The Ohio State University TBI Identification.^6^ Each PCE was then assessed via a validated retrospective concussion diagnostic interview, yielding a preliminary algorithm-generated TBI diagnosis (no mTBI, mTBI with posttraumatic amnesia [PTA], or mTBI without PTA).^25^ Each algorithm-derived determination of a TBI event was vetted and compared to the full interview free text and medical record information by experts on the study team to determine whether the PCE would be classified as a TBI. After the identification of TBI events, each event was further categorized into 3 subcategories related to timing: (1) events occurring before military service (pre–military service), (2) events that occurred during military service but not when deployed (non–deployment related), and (3) events that occurred during combat active deployment (deployment related).

### Contact Sport Participation

The history of contact sport participation was collected using the Boston University Contact Sport History Questionnaire. Individuals were classified as having a history of contact sport participation if they reported any participation in American football, basketball, boxing, cheerleading, field hockey, ice hockey, lacrosse, martial arts, rodeo, rugby, skiing, snowboarding, soccer, ultimate frisbee, water polo, and/or wrestling. Years of contact sport exposure were calculated by subtracting the age at which the individual started participating in that sport from the age at which the participant reported no longer participating in that sport. Each participant's total number of years of contact sport participation was then summed across all sports to calculate a cumulative years of contact sport exposure value (Appendix Table A1).

### Covariates

#### Demographic Data

Demographic information including age, sex, race, ethnicity, marital status, education attainment, highest rank, and branch of military service was self-reported using standardized participant intake forms. Because of the sample distribution, participants were categorized into 1 of 3 racial identity groups (non-Hispanic White, Black or African American, or other racial identity[ies]) for analyses; non-Hispanic White race was used as the referent group. For education level, approximately 88% of the sample had completed some college or above, so participants were separated into 2 groups: those who completed at least some college and those with no college education.

#### Military Deployment History

Deployment history was taken from the military status and mental health survey. This survey requested that participants report the total months of combat-related deployment that they experienced for each individual deployment experience. Total combat-related deployment time was then calculated by summing the total number of months of combat-related deployment reported across all deployments.

### Statistical Analysis

Demographic information, mTBI history (yes vs no), total number of mTBI events in specific discrete subcategories (lifetime number, before military service, non–deployment related, and deployment related), and military deployment history were evaluated using descriptive statistics. These included mean (standard deviation) or median (interquartile range) for continuous measures and number (percentage) for categorical measures. Between-group differences (contact sport history vs no contact sport history) were subsequently compared using the *t* test or Wilcoxon rank-sum test for continuous variables and the Pearson chi-square test or Fisher exact test for categorical measures.

To estimate the association between binary outcomes of mTBI events (yes vs no) and contact sport participation history (yes vs no) and contact sport duration, we first fit a set of logistic regression models for any lifetime mTBI events and each mTBI subcategory separately. We then fit a second set of logistic regression models to predict the outcomes listed above by 4 categorical levels of contact sport exposure years (0, 1-5, 6-10, and >10 years). We reported the odds ratios, 95% confidence intervals, and *P* values for each model. Predictors were considered statistically significant if the 95% confidence interval did not contain the number 1.

To further explore the relationship between the total number of mTBI events and contact sport participation history (yes vs no) or exposure duration, we fit a negative binomial regression model for the lifetime total number of mTBI events and for the number of events in each mTBI subcategory separately. We first predicted the number of events by contact sport participation (yes vs no) and then by 4 categorical levels of contact sport exposure years (0 [referent], >0 to 5, >5 to 10, and >10 years). We calculated incidence risk ratios (IRRs) and accompanying 95% confidence intervals for each predictor along with *P* values for each model. Predictors were considered statistically significant if we observed a result with a 95% confidence interval that did not include 1.0.

After each of the initial unadjusted models, follow-up models were then fit while adjusting for age, sex, race, ethnicity, marital status, and education (model 2), and then fit again while adjusting for service rank, military branch, and military deployment time (model 3). All analyses were performed using Stata MP 18.0 (StataCorp).

## Results

In our analytic sample of 2651 servicemembers and veterans taken from the LIMBIC-PLS database, 62% (n = 1643) reported a history of contact sport participation (Table 1). There were significant differences between individuals with and without a history of contact sport participation for sex, ethnicity, marital status (married vs not married), education, military rank, and combat-related deployment time. Specifically, individuals with a history of contact sport participation were more likely to be male, non-Hispanic, married, have attained some college education or more, and be classified as an officer (or warrant officer) than those without a history of contact sport participation. Additionally, individuals with a history of contact sport participation reported greater durations of combat-related deployment (18 ± 5 vs 15 ± 7 months).

**Table 1:** Veterans’ Characteristics Stratified by Those With and Without a History of Contact Sport*^a^*

	Total	No Contact Sport	Contact Sport	*P* Value
	(N = 2651)	(n = 1008)	(n = 1643)	
Age, y, mean (SD)	42.7 (10.6)	42.4 (11.3)	42.9 (10.2)	.31*^b^*
Sex				<.001*^c^*
Male	2325 (88.0)	824 (81.7)	1501 (91.4)	
Female	326 (12.0)	184 (18.3)	142 (8.6)	
Race				.71*^c^*
White	1905 (71.9)	726 (72.0)	1179 (71.8)	
African American	481 (18.1)	187 (18.6)	294 (17.9)	
Other/unknown	265 (10.0)	95 (9.4)	170 (10.3)	
Ethnicity				<.001*^c^*
Not Hispanic	2194 (82.8)	788 (78.2)	1406 (85.6)	
Hispanic	457 (17.2)	220 (21.8)	237 (14.4)	
Marital status				<.001*^c^*
Unmarried/divorced/separated/others	1034 (39.0)	440 (43.7)	594 (36.2)	
Married	1617 (61.0)	568 (56.3)	1049 (63.8)	
Education				<.001*^c^*
Below college	323 (12.2)	163 (16.2)	160 (9.7)	
Some college or above	2328 (87.8)	845 (83.8)	1483 (90.3)	
Rank				<.001*^c^*
Enlisted	2160 (81.5)	870 (86.3)	1290 (78.5)	
Officer	491 (18.5)	138 (13.7)	353 (21.5)	
Branch of service				.09*^d^*
Air Force	271 (10.2)	99 (9.8)	172 (10.5)	
Army	1692 (63.8)	667 (66.2)	1025 (62.4)	
Marines	405 (15.3)	134 (13.3)	271 (16.5)	
Navy	271 (10.2)	101 (10.0)	170 (10.3)	
Others	12 (0.5)	7 (0.7)	5 (0.3)	
Combat-related deployment, mo				
Median (IQR)	18.0 (12.0-29.0)	15.0 (11.0-27.0)	18.0 (12.0-30.0)	<.001*^e^*
Categorized by 4 levels				
Mean (SD)	21.9 (16.1)	20.2 (14.9)	22.9 (16.8)	
<12	**651** (24.5)	280 (27.8)	371 (22.6)	
≥12 to <18	674 (25.4)	277 (27.5)	397 (24.2)	
≥18 to <30	701 (26.4)	243 (24.1)	458 (27.9)	
≥30	625 (23.6)	208 (20.6)	417 (25.4)	
Years of contact sports, mean (SD)	12.4 (12.5)	0.0 (0.0)	12.4 (12.5)	
Years of contact sports, median (IQR)	8.0 (4.0-17.0)	0.0 (0.0-0.0)	8.0 (4.0-17.0)	
Years of contact sports				
0	1008 (38.0)	1008 (100.0)	0 (0.0)	
>0 to 5	576 (21.7)	0 (0.0)	576 (35.1)	
>5 to 10	376 (14.2)	0 (0.0)	376 (22.9)	
>10	691 (26.1)	0 (0.0)	691 (42.1)	
Start contact sport before military				
No	1045 (39.4)	1008 (100.0)	37 (2.3)	
Yes	1606 (60.6)	0 (0.0)	1606 (97.7)	

a Data are presented as n (%) unless otherwise indicated.

b *t* test.

c Chi-square test.

d Fisher exact test.

e Wilcoxon rank-sum test.

In total, 82% (n = 2172) had a history of at least 1 lifetime mTBI event (Table 2). Participants with a history of contact sport participation demonstrated a higher cumulative incidence of mTBIs across several contexts compared to similar peers without contact sport history. While the difference in the overall prevalence of lifetime TBI (yes vs no) was modest (83% with vs 80% without contact sport participation), there were more pronounced differences for mTBI before military service (55% vs 43% for those without). Similar patterns emerged for non–deployment-related mTBIs, with 70% of the contact sport group reporting at least 1 event compared to just 60% in the noncontact sport group. In contrast, no difference was observed between the groups in the proportion reporting deployment-related mTBIs.

**Table 2:** Lifetime mTBI History*^a^*

	Total	Without History of Contact Sport	With History of Contact Sport	*P* Value
	(N = 2651)	(n = 1008)	(n = 1643)	
Any mTBI (yes vs no)	2172 (81.9)	804 (79.8)	1368 (83.3)	.023*^b^*
No. of mTBIs	2.0 (1.0-3.0)	2.0 (1.0-3.0)	2.0 (1.0-3.0)	<.001*^c^*
Any non–deployment-related mTBI (yes vs no)	1759 (66.4)	601 (59.6)	1158 (70.5)	<.001*^b^*
No. of non–deployment-related mTBIs	1.0 (0.0-2.0)	1.0 (0.0-2.0)	1.0 (0.0-2.0)	<.001*^c^*
Any deployment-related mTBI (yes vs no)	1421 (53.6)	529 (52.5)	892 (54.3)	.36*^b^*
No. of deployment-related mTBIs	1.0 (0.0-1.0)	1.0 (0.0-1.0)	1.0 (0.0-1.0)	.29*^c^*
Any mTBI before military service (yes vs no)	1342 (50.6)	434 (43.1)	908 (55.3)	<.001*^b^*
No. of mTBIs before military service	1.0 (0.0-1.0)	0.0 (0.0-1.0)	1.0 (0.0-2.0)	<.001*^c^*

a Data are presented as n (%). mTBI, mild traumatic brain injury.

b *t* test.

c Chi-square test.

Participants with a history of contact sport participation were 1.26 (95% CI, 1.03-1.54; *P* = .023) times as likely to have sustained at least 1 mTBI before military service and were also more likely to have experienced a non–deployment-related mTBI event than those who reported no contact sport participation (OR, 1.62; 95% CI, 1.37-1.91; *P* < .001). In contrast, there was no difference for deployment-related mTBI between the groups (Table 3). The lifetime rate of mTBIs among individuals with a contact sport history was 1.22 (95% CI, 1.14-1.30; *P* < .001) times the rate for individuals without such a history (Table 4). Moreover, the contact sport group also had higher rates of non–deployment-related mTBIs (IRR, 1.33; 95% CI, 1.22-1.45; *P* < .001) before military service. These findings remained significant after adjusting for demographic factors (age, sex, race, ethnicity, marital status, and education [model set 2]) and military history (rank, branch, and years of combat-related deployment [model set 3]).

**Table 3:** Odds Ratio of mTBI Events for the History of Contact Sport (N = 2651)*^a^*

	Model 1		Model 2		Model 3	
	OR (95% CI)	*P* Value	OR (95% CI)	*P* Value	OR (95% CI)	*P* Value
Any mTBI (yes vs no)	1.26 (1.03-1.54)	.023	1.13 (0.92-1.39)	.24	1.13 (0.91-1.39)	.26
mTBI non–deployment related (yes vs no)	1.62 (1.37-1.91)	<.001	1.49 (1.26-1.76)	<.001	1.48 (1.25-1.76)	<.001
mTBI deployment related (yes vs no)	1.08 (0.92-1.26)	.36	1.04 (0.88-1.23)	.64	1.02 (0.86-1.20)	.86
mTBI before military service (yes vs no)	1.63 (1.39-1.91)	<.001	1.48 (1.26-1.75)	<.001	1.50 (1.27-1.76)	<.001

a mTBI, mild traumatic brain injury.

**Table 4:** IRR of Number of Lifetime mTBI Events (N = 2651)*^a^*

	Model 1		Model 2		Model 3	
	IRR (95% CI)	*P* Value	IRR (95% CI)	*P* Value	IRR (95% CI)	*P* Value
Total No. of mTBI	1.22 (1.14-1.30)	<.001	1.16 (1.08-1.24)	<.001	1.15 (1.07-1.23)	<.001
No. of mTBI non–deployment related	1.33 (1.22-1.45)	<.001	1.27 (1.16-1.38)	<.001	1.25 (1.15-1.37)	<.001
No. of mTBI deployment related	1.06 (0.96-1.17)	.25	1.02 (0.93-1.12)	.67	1.01 (0.92-1.11)	.88
No. of mTBI before military service	1.44 (1.29-1.60)	<.001	1.34 (1.20-1.49)	<.001	1.34 (1.20-1.49)	<.001

a IRR, incidence rate ratio; mTBI, mild traumatic brain injury.

**Figure 1.**
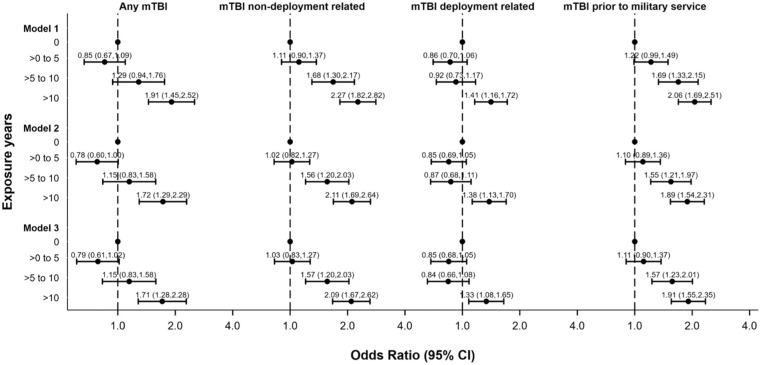
Odds ratio of mild traumatic brain injury (mTBI) events for exposure years (N = 2651).

**Figure 2.**
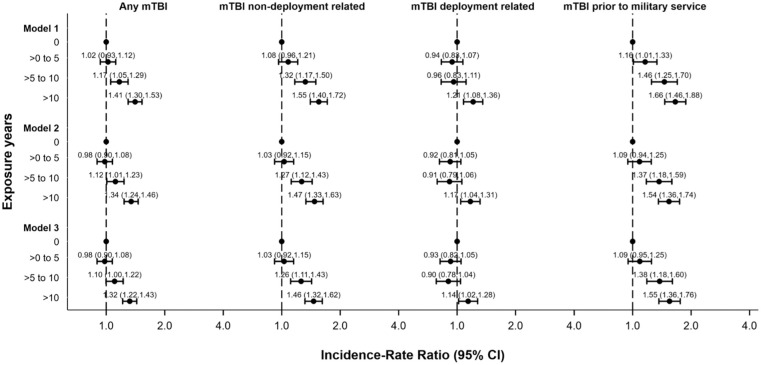
Incidence rate ratio of number of mild traumatic brain injury (mTBI) events (N = 2651).

A clear dose-response relationship was observed for the likelihood of experiencing an mTBI and duration of premilitary contact sport participation (Figure 1). The odds of experiencing an mTBI were higher in relation to longer durations of contact sport participation. Notably, for those with extensive contact sport participation (>10 years), the likelihood of having previously experienced an mTBI was nearly doubled (OR, 1.91; 95% CI, 1.45-2.52) compared to individuals without contact sport participation. Overall, mTBI events occurred more frequently among individuals with longer periods of contact sport exposure (IRR, 1.41; 95% CI, 1.30-1.53; *P* < .001) (Figure 2).

## Discussion

The goal of this study was to explore the associations of contact sport participation and lifetime TBI exposure in combat-exposed servicemembers and veterans to better understand the cumulative effect of high-risk exposure, starting with participation in contact sports and subsequently combined with military service. In support of our hypothesis, we found that individuals with a history of contact sport participation had higher odds of having sustained any mTBI and experienced mTBI at a higher rate over their lifetime than those without a history of contact sport participation. This was most pronounced for mTBI events before military service, where individuals with a history of any contact sport across all contact sport duration exposure levels were more likely than peers without a contact sport history to have experienced ≥1 mTBIs before military service. These individuals also experienced mTBI events at a higher rate before military participation than those without contact sport exposure. Moreover, a consistent trend in an increase in odds of experiencing an mTBI was observed as the total number of years of contact sport participation increased. Of note, the only association we found between contact sport history and risk of deployment-related mTBI was among participants with >10 years of contact sport participation. However, our overall results show that, independent of an individual's age, sex, race, ethnicity, marital status, education level, military rank, and branch of the military, individuals with a history of contact sport participation have a higher likelihood of experiencing an mTBI and a higher number of mTBIs across their lifetime.

When exploring the odds of deployment-related and non–deployment-related mTBIs, the most vulnerable group was those with the highest level (>10 years) of contact sport exposure. Individuals with >10 years of contact sport exposure were more likely to have experienced deployment and non–deployment-related mTBIs and experienced mTBIs at a greater rate than those with limited and no contact sport exposure. One potential explanation for the observed higher odds of mTBI across all 3 measured exposure periods (premilitary, deployment, and nondeployment) is increased susceptibility to future mTBIs after an initial mTBI. This explanation is supported by reports that have described higher odds of experiencing a subsequent mTBI among individuals with ≥1 previous mTBIs such that individuals with a previous injury are 1 to 3 times more likely to experience a future injury, and individuals with ≥3 previous injuries may be up to 5 times more likely to experience a future injury.^1,11,28^ Therefore, those individuals with previous contact sport participation may have had additional exposures to repetitive head impacts and previous injury that may have predisposed them to future injury throughout adulthood and military service. Furthermore, age of first exposure to contact sport participation has been correlated with higher likelihood of experiencing an mTBI, with an observed 5% increase in likelihood for each year of exposure.^2^ Therefore, individuals with >10 years of contact sport exposure may have higher vulnerability to subsequent injuries. In the present study, the contact sport group had a mean of 12.4 years of contact sport participation, suggesting that those with an above-average amount of contact sport exposure may be at a higher risk of experiencing ≥1 brain injuries coming into their military service and throughout military service, as exposure to potentially concussive events began at a younger age. It is unknown whether these individuals are more susceptible to mTBI from a given traumatic force, or they are being placed or placing themselves in harm's way more often, leading to more exposures, or both. Alternatively, prior experience with an injury may improve recognition and reporting behaviors for subsequent injuries, but these factors were not measured in the present study.

To adjust for potential confounding factors, we considered numerous covariates that have been previously associated with mTBI rates in both athletes and military personnel (age, sex, race/ethnicity, and education level).^13,14^ In our adjusted models, we observed small differences in the odds of experiencing an mTBI among contact sport participants after controlling for age, sex, race, ethnicity, marital status, and education. However, similar differences were not observed in relation to IRRs, which accounted for the years of at-risk exposure. Previous research has demonstrated that female athletes as well as racial minorities experience concussions at a higher rate than their male and/or White-identifying counterparts in comparable sports and military occupational specialties.^3,13,14,24^ Moreover, age has previously been positively correlated with a higher probability of previous brain injury.^24^ In the present study there was a significant difference between contact sport participation with regard to the distribution of sex in each group, but no differences in racial/ethnic representation were observed between groups. Moreover, there was no difference in the mean age between groups.

Further research is necessary to explore the big-picture implications of an increased mTBI burden experienced by military personnel who participate in contact sports before and throughout military service; it should also consider the potential protective effects of sport participation on holistic long-term health and well-being, as demonstrated by previous research. Of particular interest are those individuals with extensive contact sport participation (>10 years) before military service. While mounting evidence has described potential cumulative neurological consequences such as depression,^10^ cognitive impairment,^9,18^ neurodegenerative disease,^8^ posttraumatic stress disorder,^21,22^ decreased neuropsychological performance,^11^ and chronic traumatic encephalopathy^16,23^ in individuals with a history of mTBI, other research has found limited evidence of such profuse findings in individuals with mTBI history and suggested that these findings may be a result of study design limitations, purposive or convenience sampling, or other biases. To continue this work, researchers should begin to explore research that attempts to consider both the potential positive protective effects, such as increased cardiovascular function, life satisfaction, and well-being, previously demonstrated and the potential detrimental effects of cumulative exposure to head impacts across a lifespan. Moreover, future research should consider how to optimize the baseline for this group to assist in optimizing treatment and return to baseline measures for military servicemembers who experience recurrent mTBIs.

The present study has several limitations that should be noted. First, the researchers did not include military occupational specialty or cumulative blast exposure data, which could have contributed to the observed differences between groups based on the individualized concussion risk for each occupational specialty. Similarly, no data on sport position, playing time, practice patterns, and so forth, were included that might lend a fuller picture of PCE exposure compared to years of engagement. We additionally did not account for potential effects of participating in limited-contact or noncontact sports, which might also expose participants to incidental impact exposures. Moreover, the LIMBIC-CENC PLS cohort who consented for longitudinal research study participation may not be representative of the entire servicemember and/or veteran population.

## Conclusion

Our study brings to light several relevant topics that may continue to shape the way servicemembers and veterans are screened for mTBI history. Our findings showed that individuals who participated in contact sports were more likely to sustain a pre–military service mTBI as well as non–deployment-related mTBI events while in the military and after military service. There was no difference in the likelihood of a deployment-related mTBI, however. Lastly, those with >10 years of premilitary contact sport participation had the highest odds of sustaining an mTBI throughout pre–military service, deployment-related, and non–deployment-related epochs. As such, it may be valuable to incorporate mTBI history into the screening of servicemembers before their military service. Special attention may also need to be paid to those reporting >10 years of previous contact sport participation, as they may be at the highest risk for future brain injury.

## Appendix

**Appendix Table A1:** Sport Types for the Contact Sport

Sports Type	Frequency	Percent	Cumulative
Skiing, downhill	10	0.29	0.32
Basketball	562	17.92	18.23
Boxing	254	8.1	26.33
Cheerleading	21	0.67	27
Diving	3	0.1	27.1
Extreme sports	7	0.22	27.32
Field hockey	8	0.26	27.57
Gymnastics	9	0.29	27.86
Hockey	132	4.16	32.04
Lacrosse	52	1.66	33.73
Martial arts	142	4.53	38.25
Other contact	2	0.06	38.32
Rodeo	6	0.19	38.51
Rugby	90	2.87	41.38
Soccer	458	14.6	56.01
Tackle football	1114	35.51	91.52
Team handball	1	0.03	91.55
Ultimate frisbee	8	0.26	91.81
Water polo	12	0.38	92.19
Wrestling	245	7.81	100
Total	3136	100	
